# Medical College of Philadelphia

**Published:** 1840-05-11

**Authors:** 


MEDICAL COLLEGE OF PHILADELPHIA.

In conformity with the provisions of the charter of incorporation of the Medical College of Philadelphia, the following officers have been duly elected by the College:—

*President.*—Thomas T. Hewson, M. D.
*Vice Presidents.*—Thomas Harris, M. D. Charles D. Meigs, M. D.
*Treasurer.*—Henry Bond, M. D.
*Corresponding Secretary.*—John Bell, M. D.
*Curator.*—Joseph Brookfield, M. D.
*Recording Secretary.*—Joseph Warrington, M. D.

*Board of Examiners.*

Jacob Randolph, M. D., Charles D. Meigs, M. D., for one year.

D. F. Condie, M. D., Robert Bridges, M. D., for two years.

Reynell Coates, M. D., Caspar W. Pennock. M. D., for three years.

